# The Effect of a Direct Fed Microbial on Liveweight and Milk Production in Dairy Cattle

**DOI:** 10.3390/ani14071092

**Published:** 2024-04-03

**Authors:** Orlando Ramirez-Garzon, John I. Al-Alawneh, David Barber, Huanle Liu, Martin Soust

**Affiliations:** 1 Terragen Biotech, Pty Ltd., Coolum Beach, QLD 4573, Australia; msoust@me.com; 2 GCP Veterinary Epidemiology Consulting, Pty Ltd., Brisbane, QLD 4069, Australia; jalawneh@gmail.com; 3 School of Veterinary Medicine, Murdoch University, Murdoch, WA 6150, Australia; 4 DairyNEXT Nutrition Consulting Services, Marburg, QLD 4346, Australia; qlddairynutrition@hotmail.com; 5 Accuredit Therapeutics, Suzhou 215000, China; elzedliu@gmail.com

**Keywords:** dairy cows, direct-fed microbial, liveweight, milk yield

## Abstract

**Simple Summary:**

This study aimed to assess the impact of supplementing *Lacticaseibacillus*- and *Lentilactobacillus*-based direct fed microbials (DFMs) on dairy cow productivity in commercial settings. Two groups of 75 cows each were selected from a milking herd and managed separately. Both groups were fed the same diet, but one group received a daily supplement of DFM top dressed on the feed. The cows that received the DFM supplement showed increased liveweight mobilization and produced more milk, especially during early lactation.

**Abstract:**

This longitudinal study aimed to quantify the effects of dietary supplementation of a direct-fed microbial (DFM) consisting of three lactobacilli isolates on milk yield, milk fat and protein yields, somatic cell count (SCC), and liveweight in a single dairy herd in Australia. A total of 150 dairy cows were randomly selected based on parity and days in milk and divided into two groups: control (*n* = 75) and DFM treatment (*n* = 75). Throughout the study, the two groups of cows were housed separately in a dry lot yard, and each group had their own feeding area. For the DFM treatment group, selected cows in mid-lactation were supplemented with 10 mL/cow/day of the DFM via top dressing of the feed for the remainder of the lactation and through the dry period, extending into subsequent lactation. The control group had no supplementation. The milk yield and liveweight were recorded daily. Milk samples were collected every two months for milk component analysis (fat, protein, and somatic cell count [SCC]). The DFM-treated cows gained more liveweight across the study (19.40 kg, 95% CI 0.44 kg; 38.30 kg, *p* = 0.05) compared to the control cows. In the second production year, the DFM-treated cows mobilized more liveweight (−6.06 kg, 95% CI −10.49 kg; −1.61 kg, *p* = 0.01) and produced more milk (0.39 L/d 95% CI 0.10; 0.89, *p* = 0.05). Over a full lactation, DFM cows yielded at least 258 L (95% CI 252 L; 265 L) more milk than controls. No significant differences were found in fat and protein yield or SCC. This study suggests that consistent and ongoing supplementation with a *Lacticaseibacillus*- and *Lentilactobacillus*-based DFM could have a positive effect on milk production, but further research is needed to understand the underlying mechanism.

## 1. Introduction

Growing concerns about the excessive use of antimicrobials in livestock production for therapeutic, metaphylactic, and prophylactic purposes have prompted the search for natural and safe alternatives in livestock production systems [1]. Currently, despite the trends towards increased regulations on the use of antimicrobials worldwide, including the legally imposed discouragement and limitations in many regions, they are still utilized in food animal production as prophylactics to improve animal health and productivity [2]. In 2020, global antimicrobial usage (AMU) for livestock was estimated at 99.5 tons of active ingredients, and a further 8.0% increase to 107.4 tons is projected by 2030, predominantly influenced by patterns of use in Asia/Oceania and the Americas [3]. 

Alternatives are being investigated to help reduce the use of antimicrobials, without reducing farm productivity and profitability. One such alternative is the use of direct-fed microbials (DFMs), which are feed supplements containing live microorganisms (bacteria and fungi) that can be orally administered to produce a beneficial health response in the host animal [4,5,6]. Commonly used strains include those in the genera of the former *Lactobacillus*, *Bifidobacterium*, *Enterococcus*, *Streptococcus*, *Bacillus*, and *Propionibacterium*, as well as fungal species like *Aspergillus* and *Saccharomyces*, which have gained popularity in the livestock industry. DFMs function within the rumen as either lactic acid-producing bacteria (LAB) or lactic acid-utilizing bacteria (LUB) [5], with strains in the former *Lactobacillus*, *Bifidobacterium*, and *Saccharomyces* being the most widely used [6,7]. 

The use of DFMs during lactation and the transition periods is a promising strategy to enhance the performance and health of adult dairy cattle [8,9]. DFMs have the potential to modify the ruminal microbiome [10,11], alter volatile fatty acids (VFA) metabolism [6,11,12], and reduce intestinal infections by colonizing the digestive tract and exhibiting antimicrobial activity against enteric pathogens [13]. Additionally, DFMs have demonstrated the ability to affect enteric methane emissions [14] and enhance milk quality [15] and milk components [12,16]. However, the effects of supplementing DFMs on milk production have yielded contradictory findings. While some studies have reported improvements in milk yield [8,9,15] and milk components [15,17], others have found no significant enhancements in milk production [10,18,19] or limited improvements restricted to health related aspects such as reducing the risk of ruminal acidosis [20], lowering the incidence of metabolic diseases (i.e., subclinical ketosis [21]), and lowering the somatic cell count (SCC) in milk [15]. These discrepancies in outcomes could be due to several factors, including the specific strain of DFM employed, whether it is a DFM comprising a single or a combination of multiple strains, the dosage, the duration of supplementation, and the composition of the diet, as well as intrinsic cow factors such as age, health status, rumen microbiome composition, and physiological stage.

The value of DFMs as a feed additive in dairy farming remains uncertain, as their prolonged effects are poorly understood [17]; moreover, there is a lack of clarity on how management practices like dietary changes (i.e., from lactation diet to dry period diet) might impact their efficacy. Although some studies have assessed the effects of DFM supplementation over short periods of time, ranging from a few weeks [10,17,19] to 5–7 months [16], within the same lactation, few have investigated the potential carryover effects of DFMs on milk yield and composition during subsequent lactations. 

Bacteria from the genus formerly known as *Lactobacillus*, an important group of microorganisms used in DFM supplementation, have demonstrated an impact on the ruminal environment, resulting in increased milk yield and quality [9,14]. Thus, the present study aimed to evaluate the effects of supplementing the diet with a consortia of three species of live bacteria, *Lacticaseibacillus casei*, *Lacticaseibacillus paracasei*, and *Lentilactobacillus buchneri*, which were formerly known as *Lactobacillus casei*, *Lactobacillus paracasei*, and *Lactobacillus buchneri* respectively, on milk yield, milk fat and protein yield, and SCC, during two consecutive lactations in dairy cows.

## 2. Materials and Methods

### 2.1. Study Location and Herd

This longitudinal study was conducted at a commercial dairy farm located in Harrisville, Queensland, Australia, from September 2021 to January 2023. The milking herd consisted of approximately 350 Holstein cows from which the study cows were randomly selected. The study cows were housed and managed as two separate groups, including during feeding (mixed ration fed on a feedpad and grazing pasture) and milking. The feeding system was classified as a partial mixed ration (PMR) consisting of a mixed ration offered on a covered feedpad within a dry lot during the day and grazing pasture at night. The mixed ration consisted predominantly of maize silage or barley silage, lucerne hay, soybean silage, canola meal, barley, or wheat grain, with 1.5 kg of barley or wheat grain fed twice daily in the dairy. The pasture intake was up to 6 kg of ryegrass or kikuyu pasture grazed during winter and summer, respectively. Both groups were housed in the same dry lot, with separate feeding and loafing areas adjacent to each other when offered the PMR, with free access to water. Each group was offered the ration once daily at 0600 in their own feeding area. The length of the feedpad was 70 m, which provided sufficient space (0.9 m per cow) for all cows to consume the feed [22]. When grazing pasture, the paddock was split into two strips longitudinally, with each treatment group randomly allocated a strip of pasture and grazed separately. 

The total diet was balanced with available ingredients and variable pasture quality to meet the milk production targets based on the lactation stage throughout the study (Table 1). The mixed ration was provided once a day to both groups with a target dry matter intake (DMI) of 21 to 22 kg/cow/day, including pasture intake. Cows in the DFM group were supplemented with 10 mL/cow/day of a DFM top dressed on their mixed ration. This supplementation occurred from mid-lactation in the first production year through to the end of lactation, extending across both the dry period and subsequent lactation in the second production year. Administration was carried out using a 2 L manual pressure sprayer (245 kPa maximum pressure, Aqua Systems Australia). The DFM contained a total of approximately 3.5 × 10^9^ CFU/mL in a consortium of three strains of live bacteria, *L. buchneri* Lb23, *L. casei* Lz26, and *L. paracasei* T9 [14].

### 2.2. Study Animals

A total of 150 primiparous and multiparous Holstein cows were randomly selected from the milking herd based on parity and days in milk (DIM). The cows were randomly assigned into two groups (control; *n* = 75 and DFM; *n* = 75) mid-way through their lactation in the first production year. Thirty-seven percent of the study animals were in first lactation, 27% in second lactation, and 36% in third lactation. The study cows averaged 3.6 years (standard deviation (SD) ±1.1 years), 2.0 (±0.8) parities, 31 (±9.1) L/cow/d, 127 DIM (±55), and 590 kg (±67) liveweight (LW). For this study, the primary outcome was milk production; therefore, a minimum of fifty cows in each of the experimental groups enabled the detection with 95% confidence, with a power of 80%, and a pooled variance of 12 L, a difference in mean milk production between the experimental groups of 2 L (95% confidence interval for differences between means = 0.60 to 3.40 L). The cows were studied across two production years: the first production year (2021/2022) from approximately mid-lactation until calving, and the second production year (2022/2023) from calving until late lactation.

The cows were milked twice daily at 0400 h and 1500 h, and the individual daily milk yield was electronically measured using the herd management software DairyPlan C21A (GEA Farm Technologies, Bönen, Germany). The daily milk yield was aggregated into weekly averages for statistical analysis. Composite milk samples were collected approximately every 6 to 8 weeks, as part of the herd’s udder health and mastitis control management strategy, and to assess milk fat and protein yield and somatic cell count. Milk samples were collected using on-farm automated milk sampling meters (GEA Westfalia, Melbourne, Australia). The milk samples were placed into vials containing a milk preservative (Bromoponol, Novachem, Heidelberg, Australia), and were shipped to a commercial analysis laboratory (Dairy Express Herd Recording Service, Armidale, NSW, Australia). Cow weights were recorded automatically after each milking session using a walk-over weigh (WOW) unit (Datamars, Brisbane, Australia). 

### 2.3. Statistical Analysis and Data Management

All data analyses were carried out in R (R Development Team, 2023).

Each cow’s LW change (dLW) was calculated as ‘LW on week’ minus ‘Average LW at baseline’ (baseline is defined as two weeks before study start date). The milk SCCs were log transformed to stabilize the variance and to meet the assumption of normality distribution. There were two final datasets: the weekly milk and LW, and every second month milk components datasets. The weekly milk and LW dataset comprised the unique cow identification number, the cow experimental group (Control and DFM), production season (categorical variable with two levels), cow parity (categorical variable with two categories: 1 for first and second parity cows; and 2 for 3rd or more parity cows), days in milk (DIM), cow dLW (continuous variable; kg), breed (Holstein-Friesian [HF] or HF-cross), average milk production at baseline (L), and average LW at baseline (kg). The milk component dataset comprised the cow identification number, experimental group, parity, DIM, breed, dLW, LW at baseline, milk yield (kg), milk fat yield, and milk protein yield (kg) at baseline, milk somatic cell counts (SCCs, 1000 cells/mL), and SCCs at baseline.

The mean differences in milk yield, milk fat yield, milk protein yield, SCC, and dLW for both experimental groups were compared using separate linear mixed-effects regression models, which allowed for clustering within cows, clustering within the experimental group, and repeated measures over time. In these models, the cow was fitted as a random effect and the experimental group was fitted as a fixed effect.

The following modeling process was used to develop the milk yield model, which applies to all remaining models. To estimate the effect of cows’ experimental group on milk yield, a linear mixed effect regression model was fitted to estimate milk yield as a function of a set of explanatory variables (i.e., DIM, milk yield at baseline, experimental group, breed, production season, and parity). The model was built using a forward modeling procedure, with cow fitted as a random intercept and DIM fitted as a random slope, to account for the repeated measure within cow. The error terms of the residuals were assumed to follow a normal distribution with a mean of zero and variance of ***σ^2^***, and followed an autoregressive correlation structure of the first order. First-order interaction terms were tested and were retained if the interaction terms were significant at a likelihood ratio test *p*-value of 0.05 or less. Except for experimental groups and their first-order interaction with DIM, explanatory variables were retained in the final model if they achieved statistical significance at a likelihood ratio test *p*-value of 0.05 or less. The order of DIM polynomial (if any) was determined by the likelihood ratio test and the Akaike information criterion (AIC). The overall model fit was based on the AIC and visual assessment of *Pearson’s* residuals against fitted values, and Q-Q standardized residuals against standardized normal quantiles to test the assumption of homogeneity of the variance of the error terms. All analyses were carried out using nlme [23] and lme4 [24] statistical packages in R.

## 3. Results

A total of 9253 individual weekly milk yield and LW records and 717 milk component records were available for the analyses. A total of 122 cows (81%) completed the study, with 66 cows (88%) in the control group and 56 cows (75%) in the DFM treatment group. Farm management chose to cull 28 cows from the study for the following reasons: mastitis, *n* = 7; reproductive performance, *n* = 13; lameness *n* = 3; low milk production, *n* = 3; and traumatic reticuloperitonitis, *n* = 2. 

### 3.1. Milk Yield and Liveweight

**Figure 1:**
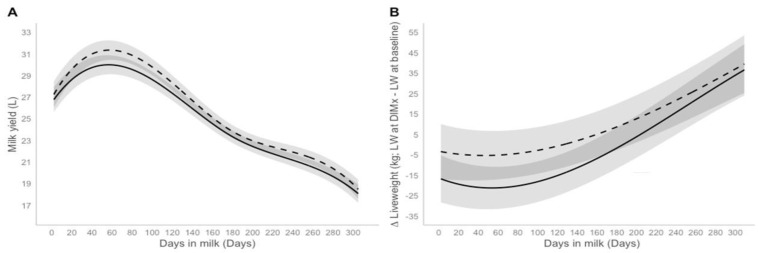
Line plot of predicted marginal milk yield means (**A**), change in liveweight (**B**) and their 95% confidence intervals of parity 1 category and parity 2 category in the control group (solid line) and the DFM Group (dashed line) that calved in the 2022/2023 production year and had an average milk production at baseline of 25 L/d and an average liveweight at baseline of 550 kg.

During the 2021/2022 production year, the control cows yielded more milk on average than the DFM-treated cows, 33 L (±7) vs. 32 L (±8), respectively. The DFM-treated cows were supplemented from 135 (±54) DIM until calving during the first production year, and from calving until 208 (±94) DIM in the second production year (Table 2). However, in the second production year, on average the DFM-treated cows produced more milk (0.39 L/d 95% CI 0.10 L; 0.89 L, *p* = 0.05; Table 3) compared with the control cows. After controlling for the effect of variables in Table 3, on average, the predicted milk yield for the DFM-treated cows across the full lactation was at least 258 L (95% CI 252 L; 265 L, *p* = 0.05) greater than the control cows (Figure 1A).

In the second production year, the DFM-treated cows, on average, gained more liveweight (19.40 kg 95% CI 0.44; 38.30, *p* = 0.05) throughout the study compared to control cows (Figure 1B). In the 2022/2023 production year, on average, the DFM-treated cows mobilized more LW (−6.06 kg 95% CI −10.49 kg; −1.61 kg, *p* = 0.01; Table 4) in the early stage of lactation compared to control cows.

### 3.2. Milk Components

No differences between the groups were found in the fat yield (0.04 kg 95% CI −0.22; 0.31 kg/day; *p* = 0.76; Table S1), protein yield (−0.04 kg 95% CI −0.15; 0.30; *p* = 0.53; Table S2), or log milk somatic cell counts (−0.33 95% CI −2.37; 1.68; *p* = 0.72; Table S3).

## 4. Discussion

This study aimed to evaluate the extended effects of supplementing a DFM on milk yield and components in Holstein dairy cows in a single commercial setting. While previous studies have reported potential benefits of using DFMs to enhance milk yield during the lactation period [13,15,25], in the current study there were no observed improvements in milk production following DFM supplementation of mid-lactation cows during the first production year (2021/2022). The average milk yield tended to be higher in control cows throughout this production season, although the difference was not statistically significant. This lack of improvement in milk yield, post-DFM supplementation, aligns with findings in other studies using different combinations of LABs, *Propionibacterium*, and/or yeast. Raeth-Knight et al. [18], Ferraretto and Shaver [26], and Lawrence et al. [27] did not observe significant changes in milk yield or milk composition (fat and protein) when supplementing *L. acidophilus* and *P. freudenreichii* in mid-lactation Holstein cows for 10–12 weeks. However, Kiani Asil et al. [28] reported improved feed intake and milk yield in mid-lactation crossbreed Holstein cows through supplementation of *L. fermentum* and *S. cerevisiae* over 54 days. A possible explanation for the lack of an initial response following DFM supplementation is that the mid-lactation cows were in a physiological and metabolic state that might have negatively influenced their initial responses to DFM supplementation. As cows transition from mid- to late lactation, they enhance their energy balance, undergo pregnancy, and naturally experience a reduction in milk yield [29]. Consequently, the energy demand for milk synthesis decreases compared to early lactation [30]. This observation is supported by Goetz et al. [31], who supplemented mid-lactation cows with two types of rumen microbes and found that lower producing cows increased their energy-corrected milk (ECM), while higher producing cows showed a decline in ECM and increased body condition scores over time with supplementation. These results suggest that the advantages observed in ruminal fermentation, digestibility, and energy production following DFM supplementation may be redirected towards restoring liveweight (as found in this study), building body reserves for the next lactation, and maintaining pregnancy rather than being directed towards milk production. However, Goldsmith et al. [32] supplemented cows with a consortia of native ruminal microorganisms from 90 DIM for 112 days and reported a daily weight gain of 0.19 kg/d lower in supplemented cows compared to controls, alongside decreased dry matter intake, organic matter digestibility, and plasma insulin, without observing differences in milk yield between groups. This suggests that the energy utilization may have prioritized milk production over weight gain [32,33], highlighting a more targeted re-direction of energy than previously suggested. In conclusion, the interaction between the physiological state of mid-lactation cows and the DFM supplement used in this study could influence energy partitioning by increasing body reserves at the cost of milk yield. Further investigation is needed to elucidate the underlying mechanisms.

Unlike previous studies, supplementation in the present study extended beyond the end of lactation of the first production season (2021/2022), continued throughout the dry period until calving, and into late lactation of the second production season (2022/2023). Cows receiving DFMs were supplemented from 135 ± 54 DIM until calving and continued until 208 ± 94 DIM of the subsequent lactation. This approach aimed to ensure the establishment of the DFMs in the rumen with consistent dosing, overcoming potentially low survival rates of ruminal microbes during the approximate two months supplementation gap between the dry period and the new lactation season [25]. In studies where DFM supplementation is used before calving, supplementation starts either during the dry period or during the transition period, and continues through early lactation [8,16,21,34,35]. To our knowledge, this is the first study that evaluated the extended and uninterrupted use of DFMs from the preceding lactation through the dry period and into the late stage of the subsequent lactation. Using different DFMs strains as in our study, Carpineli et al. [34] observed 2.5 kg/d higher milk production in cows receiving a yeast culture-based product (*S. cerevisiae*) from one month before calving to 50 DIM. Similarly, Nocek et al. [8] reported higher milk production (0.9 kg/d to 2.3 kg/d) when supplementing dairy cows with a combination of yeast and *E. faecium* from 21 days before calving until 70 DIM. Similarly, in this study, during the second production year, cows fed with DFMs produced more milk during the most productive stages of lactation. Specifically, DFM cows yielded between 0.10 L/d to 0.89 L/d *p* = 0.05 more milk, reflecting a 3% increase in milk production during the initial 100 DIM compared to control cows. Previous studies using bacterial consortia during early lactation have reported similar effects. Qiao et al. [36] observed an increase in 4% fat-corrected milk when early lactation Holstein cows were supplemented with *B. licheniformis* for 10 weeks. Xu et al. [15] observed an increase in average milk yield after supplementing dairy cows with a mixture of *L. casei* and *L plantarum* from 60 DIM for 30 days. 

Typically, postpartum cows mobilize adipose and muscle tissues to meet the energy demands for milk production, resulting in weight loss [37]. In the current study, the DFM-treated cows gained more liveweight across the study (19.40 kg, 95% CI 0.44; 38.30; *p* = 0.05) compared to control cows, and after calving, the DFM-treated cows mobilized more liveweight (−6.06 kg, 95% CI −10.49; −1.61 kg), and then showed faster postpartum recovery of bodyweight. Oyebade et al. [12] found no effects on milk yield or bodyweight when supplementing early lactation cows for 90 days with a mix of *L. animalis*, *P. freudenreichii*, *B. subtilis*, and *B. licheniformis*. Consistent with our findings, Stein et al. [35] supplemented dairy cows with a *Propionibacterium* strain from −2 to 30 wks postpartum, and reported accelerated recovery of week 1 bodyweight (baseline), and between 7.1 to 8.5% increases in daily fat-corrected milk, indicating that increased milk production did not affect bodyweight changes.

We can speculate that the mode of action of the DFM to increase productivity and bodyweight was likely related to changes in ruminal microbiota, favoring fermentative and beneficial bacteria, while suppressing opportunistic pathogens [15]. This adjustment influences fermentation processes, enhances digestive metabolism, and improves nutrient availability. For instance, Xu et al. (2017) demonstrated enrichment of fermentative and cellulolytic bacteria such as *Bacteroides*, *Ruminococcus*, and *Roseburia* after supplementing dairy cows with *L. casei* and *L. plantarum*, and a higher abundance of *Faecalibacterium*, which contributed to weight gain and reduced the abundance of *Firmicutes* and *Proteobacteria*. Monteiro et al. (2022) [25] also observed lower relative abundances of *Firmicutes*, *Saccharofermentans*, and *Ruminococcus* after *L. plantarum* supplementation, which was correlated with lowered NH_3_-N concentration, suggesting enhanced nitrogen captured in the rumen, improving its efficiency. Another possible mechanism to increase productivity and bodyweight after DFM supplementations is linked to increased nutrient digestibility [12,18]. So et al. [38] reported improved milk yield, enhanced dry matter and fiber digestibility, increased energy intake, higher ruminal propionate concentrations, and reduced ammonia in dairy cows supplemented with *L. casei.* In addition, changes in ruminal volatile fatty acids favoring glucogenic precursors for milk synthesis have also been associated with enhanced milk yield [8,35]. Despite the mechanism(s) involved, this study provides evidence for the potential use of *Lacticaseibacillus*/*Lentilactobacillus* DFM consortia as a supplement to increase milk production.

No significant differences in the yield of milk components (fat and protein) or SCC between the DFM and control groups were found. This result is consistent with studies that reported no effect on milk composition with the inclusion of DFM in the diet [9,16,18,31], suggesting that DFM supplementation does not affect milk composition directly.

Field studies conducted in commercial settings present challenges due to many confounders and uncontrolled factors, which may influence study results. In this study, certain limitations were present that should be considered when interpreting the results. One notable limitation was the lack of precise control over daily individual intake per animal. The DFM was top dressed on the daily ration using a manual sprayer, which did not allow for accurate monitoring of the treatment dose intake on a per-animal basis. However, alternative approaches, such as Calan gates [9], tie-stall housing [18], or individual treatment administration [35], are impractical under commercial conditions. In this case, every cow had 0.9 m of feeding space along the feedpad to have access to the supplement; therefore, the daily and continuous DFM administration would likely ensure that an effective dose per animal was received, on average, over time. An additional limitation was that 28 out of 150 (18.6%) cows were removed from the study following farm culling guidelines. The most common causes of culling were reproductive failure and mastitis (20/28; 72%), and were unrelated to the treatment. The culling rate aligned with the observed increase in culling rates for reproductive failure and mastitis in Australian dairy herds [39]. Additionally, extreme weather conditions, such as the record-breaking rainfall in 2022 [40], impacted the study and likely contributed to the higher prevalence of mastitis in the herd.

Although these limitations exist, this observational study serves as a valuable basis for generating hypotheses to be further tested under more controlled conditions, where greater control over individual intake may be achieved. Finally, although the current study has a strong internal validity, due to constraints in external validity the direct application of the study’s findings to broader herds is limited. It is essential to highlight that the conclusions drawn from this study are relevant primarily to the specific source population and other animals belonging to the target population that share analogous characteristics and are managed under similar conditions. Therefore, it is important to undertake further investigation to build on the study’s results across various settings before extending the implications to a more extensive and diverse population.

## 5. Conclusions

Under the conditions of this field trial, it was observed that the continuous and prolonged administration of a *Lacticaseibacillus*- and *Lentilactobacillus*-based DFM to lactating dairy cows was associated with positive effects on bodyweight and milk production. The improvement in milk production was most evident in early lactation. However, the mechanism(s) underlying the increase in production were not investigated in this study. Further research under more controlled conditions is needed to determine the mechanisms by which the direct fed microbials supplement could increase production.

## Figures and Tables

**Table 1:** Ingredient and chemical composition of diet (%, DFM basis).

Ingredient	Mean	Min	Max
Grain (barley, wheat, sorghum)	19.3	15.8	27.7
Protein Meal (canola meal, soybean meal)	7.7	0	17.4
Byproducts (flour bread, carrots, sweet corn waste, chickpea millrun)	14.3	3.1	35.3
Lucerne Hay	5.8	3.3	9.9
Silage (corn, barley, oats, soybean)	34.7	14.8	49.2
Pasture (kikuyu, ryegrass)	15.3	0	43
Bypass Fat	0.6	0	1.9
Minerals (macro minerals, trace minerals premix, urea, mycotoxin binder)	2.4	1.2	3.5
**Diet Composition**			
Crude Protein (CP, %)	16.9	13.8	22.8
Neutral Detergent Fiber (NDF, %)	35.9	31.2	40
Acid Detergent Fiber (ADF, %)	23.2	19.3	26.2
Non-Fibrous Carbohydrate (NFC, %)	34.3	28.1	40.2
Fat (%)	4.4	3.6	5.6
MJ ME/Kg DM	9.5	8.6	10.8
Starch (%)	23	20.1	26.5

**Table 2:** Descriptive statistics of cow records and milk yield, milk component, and liveweight (LW) measurements at baseline and the study period, stratified by experimental groups.

	Control			DFM		
	Mean (SD)	Median (Q1–Q3)	Min–Max	Mean (SD)	Median (Q1–Q3)	Min–Max
**Baseline (before start of study period)**						
Milk yield (L)	33 (7)	33 (27–38)	19, 50	32 (8)	31 (26–37)	11, 49
Days in milk (days)	131 (53)	145 (87–172)	6, 226	135 (54)	146 (88–174)	5, 423
Parity	2 (1)	2 (1–3)	1, 3	2 (1)	2 (1–3)	1, 3
Fat yield (kg)	3 (1)	3 (3–4)	2, 10	3 (1)	3 (3–4)	2, 7
Protein yield (kg)	3 (0)	3 (3–3)	3, 4	3 (0)	3 (3–3)	3, 5
Somatic cell counts (×1000 cells/mL)	70 (73)	39 (20–93)	8, 356	98 (92)	61 (31–139)	9, 354
Liveweight (kg)	582 (57)	584 (534–612)	465, 707	577 (62)	577 (524–622)	437, 733
**Second production year (2022/2023)**						
Milk yield (L)	26 (6)	25 (21–29)	10, 50	25 (6)	24 (21–29)	8, 55
Days in milk (days)	193 (98)	207 (114–266)	2, 493	208 (94)	222 (142–276)	2, 526
Fat yield (kg)	1 (0)	1 (1–1)	0, 2	1 (0)	1 (1–1)	0, 1
Protein yield (kg)	195 (451)	65 (28–163)	4, 4927	246 (561)	94 (42–207)	7, 4704
Somatic cell counts (×1000 cells/mL)	588 (62)	584 (546–624)	398, 842	591 (67)	583 (542–636)	427, 797
Liveweight change (kg)	6 (43)	3 (−23–32)	−144, 160	14 (43)	12 (−15–41)	−166, 167

**Key:** DFM Direct fed microbials; SD standard deviation; Q1–Q3 first and third quartiles; Min Minimum; Max Maximum.

**Table 3:** Estimated coefficients and 95% confidence intervals of explanatory variables influencing the association between milk yield (L/d) and experimental groups. Coefficients are estimated from a mixed effects model with random intercepts (cow) and random slopes (days in milk).

Explanatory Variable	Coefficient (SE)	95% Confidence Interval	*p*-Value *
Intercept	23.06 (1.05)	19.38; 24.16	<0.001
Days in milk (DIM) fitted as fourth-order cubic spline **			
Experimental groups			0.91
Control	Reference		
DFM	0.09 (0.83)	−1.54; 1.72	
Milk yield at First Production year	0.23 (0.03)	0.19; 0.31	<0.001
Cow parity			<0.001
1st and 2nd (category 1)	Reference		
>2nd (category 2)	1.77 (0.16)	1.54; 2.20	
Production year			<0.001
First (2021/2022)	Reference		
Second (2022/2023)	−3.59 (0.14)	−3.87; −3.30	
DIM polynomial terms × Experimental group			0.07
Experimental Group × Production year			0.05
Control × 2021/2022	Reference		
DFM × 2022/2023	0.39 (0.19)	0.10; 0.89	

DFM * Likelihood ratio *p* values; ** fourth-order cubic spline polynomial was determined by the likelihood ratio test and model AIC. DFM—Direct fed microbials.

**Table 4:** Estimated coefficients and 95% confidence intervals of explanatory variables influencing the association between change in liveweight (kg) and the experimental groups. Coefficients are estimated from a mixed effects model with random intercepts (cow) and random slopes (days in milk).

Explanatory Variable	Coefficient (SE)	95% Confidence Interval	*p*-Value *
Intercept	−57.73 (6.24)	−69.91; −45.52	<0.001
Days in milk (DIM) fitted as fourth-order cubic spline **			<0.001
Experimental groups			0.05
Control			
DFM	19.40 (9.70)	0.44; 38.30	0.05
Liveweight at First Production year	0.22 (0.03)	0.16; 0.28	<0.001
Cow parity			<0.001
1st and 2nd (Category 1)	Reference		
>2nd (Category 2)	−49.43 (3.71)	−56.72; −41.91	
Production year			<0.001
First (2021/2022)			
Second (2022/2023)	40.9 (1.58)	37.68; 44.00	
DIM polynomial terms × Experimental group			0.06
DFM × Production year			0.01
Control × 2021	Reference		
DFM × 2022/2023	−6.06 (2.27)	−10.49; −1.61	

* Likelihood ratio *p* values; ** fourth-order cubic spline polynomial was determined by the likelihood ratio test and model AIC. DFM—Direct fed microbials.

## Data Availability

Data will be available on request by contacting the author.

## Supplementary Materials

The following supporting information can be downloaded at: https://www.mdpi.com/article/10.3390/ani14071092/s1, Table S1: Estimated coefficients and 95% confidence interval of explanatory variables influencing the association between milk fat yield (kg/d) and experimental groups. Coefficients estimated from a mixed effects model with random intercepts (cow) and random slopes (days in milk); Table S2: Estimated coefficients and 95% confidence interval of explanatory variables influencing the association between milk protein yield (kg/d) and experimental groups. Coefficients estimated from a mixed effects model with random intercepts (cow) and random slopes (days in milk); Table S3: Estimated coefficients and 95% confidence interval of explanatory variables influencing the association between milk log somatic cell count (1000× cells per mL) and experimental groups. Coefficients estimated from a mixed effects model with random intercepts (cow) and random slopes (days in milk).
