# Supplementary material for: The Effect of a Direct Fed Microbial on Liveweight and Milk Production in Dairy Cattle
Source: Animals (Basel). 2024 Apr 3;14(7):1092. doi: 10.3390/ani14071092 (PMC11010996; doi:10.3390/ani14071092)
Supplement: Supplementary file 1 [file animals-14-01092-s001.zip › animals-2889684-supplementary.pdf]

Table S1. Estimated coefficients and 95% confidence interval of explanatory variables influencing the association between milk fat yield (kg/d) and experimental groups. Coefficients estimated from a mixed effects model with random intercepts (cow) and random slopes (days in milk).

Explanatory Variable	Coefficient (SE)	95% Confidence Interval	*p*-Value *
Intercept	1.08 (0.1)	0.89; 1.27	<0.001
Days in milk (DIM, polynomial) **			<0.01
1st order	0.44 (0.18)	0.08; 0.8	
2nd order	-0.23 (0.11)	-0.45; -0.01	
3rd order	-0.29 (0.15)	-0.58; 0	
4th order	-0.37 (0.12)	-0.6; -0.13	
Experimental groups			0.76
Control	Reference		
DFM	0.04 (0.14)	-0.22; 0.31	
Cow parity			<0.001
1st&2nd (category 1)	Reference		
>2nd (category 2)	0.11 (0.03)	0.05; 0.17	
Production season			<0.01
First (2021/2022)	Reference		
Second (2022/2023)	-0.13 (0.03)	-0.19; -0.08	
Experimental Group x DIM			0.71
Control x DIM	Reference		
DFM x 1st order	-0.04 (0.27)	-0.56; 0.48	
DFM x 2nd order	-0.13 (0.17)	-0.46; 0.2	
DFM x3rd order	-0.04 (0.22)	-0.47; 0.38	
DFM x 4th order	-0.05 (0.17)	-0.39; 0.29	

* Likelihood ratio *p* values; ** fourth-order cubic spline polynomial was determined by the likelihood ratio test and model AIC. DFM—Direct fed microbials.

Table S2. Estimated coefficients and 95% confidence interval of explanatory variables influencing the association between milk protein yield (kg/d) and experimental groups. Coefficients estimated from a mixed effects model with random intercepts (cow) and random slopes (days in milk).

Explanatory Variable	Coefficient (SE)	95% Confidence Interval	*p*-Value *
Intercept	0.87 (0.11)	0.34; 1.05	<0.001
Days in milk (DIM, polynomial) **			0.06
1st order	0.19 (0.1)	-0.16; 0.75	
2nd order	-0.06 (0.05)	-0.12; 0.21	
3rd order	-0.25 (0.06)	-0.07; 0.42	
Experimental groups			0.56
Control	Reference		
DFM	-0.04 (0.06)	-0.15; 0.3	
Cow parity			<0.001
1st&2nd (category 1)	Reference		
>2nd (category 2)	-0.01 (0.02)	0.03; 0.15	
Production season			<0.01
First (2021/2022)	Reference		
Second (2022/2023)	-0.13 (0.03)	-0.19; -0.08	
Experimental Group x DIM			0.65
Control x DIM	Reference		
DFM x 1st order	0.01 (0.15)	-0.83; 0.29	

DFM x 2nd order	-0.01 (0.08)	-0.35; 0.15	
DFM x3rd order	0.03 (0.09)	-0.32; 0.26	

* Likelihood ratio *p* values; ** fourth-order cubic spline polynomial was determined by the likelihood ratio test and model AIC. DFM—Direct fed microbials.

Table S3. Estimated coefficients and 95% confidence interval of explanatory variables influencing the association between milk log somatic cell count ('000 X cells per mL) and experimental groups. Coefficients estimated from a mixed effects model with random intercepts (cow) and random slopes (days in milk

Explanatory Variable	Coefficient (SE)	95% Confidence Interval	*p*-Value *
Intercept	5.54 (0.98)	3.64; 7.43	<0.001
Days in milk (DIM, polynomial) **			<0.03
1st order	-4.58 (2.07)	-8.65; -0.56	
2nd order	-0.58 (0.82)	-2.16; 1.04	
3rd order	-1.65 (1.13)	-3.87; 0.57	
Experimental groups			0.75
Control	Reference		
DFM	-0.33 (1.05)	-2.37; 1.68	
Cow parity			<0.001
1st&2nd (category 1)			
>2nd (category 2)	0.44 (0.23)	0.01; 0.88	
Production season			<0.01
First (2021/2022)	Reference		
Second (2022/2023)	-0.13 (0.03)	-0.19; -0.08	
Experimental Group x DIM			0.45
Control x DIM	Reference		
DFM x 1st order	3.01 (2.6)	-2; 8.09	
DFM x 2nd order	-0.62 (1.21)	-2.98; 1.68	
DFM x 3rd order	0.51 (1.37)	-2.14; 3.19	

* Likelihood ratio *p* values; ** fourth-order cubic spline polynomial was determined by the likelihood ratio test and model AIC. DFM—Direct fed microbials.
